# Regulatory network modelling of iron acquisition by a fungal pathogen in contact with epithelial cells

**DOI:** 10.1186/1752-0509-4-148

**Published:** 2010-11-04

**Authors:** Jörg Linde, Duncan Wilson, Bernhard Hube, Reinhard Guthke

**Affiliations:** 1 Research Group Systems Biology/Bioinformatics, Leibniz-Institute for Natural Product Research and Infection Biology-Hans-Knoell-Institute, Beutenbergstraße 11a, 07745 Jena, Germany; 2 Department Microbial Pathogenicity Mechanisms, Leibniz-Institute for Natural Product Research and Infection Biology-Hans-Knoell-Institute, Beutenbergstraße 11a, 07745 Jena, Germany

## Abstract

**Background:**

Reverse engineering of gene regulatory networks can be used to predict regulatory interactions of an organism faced with environmental changes, but can prove problematic, especially when focusing on complicated multi-factorial processes. *Candida albicans *is a major human fungal pathogen. During the infection process, this fungus is able to adapt to conditions of very low iron availability. Such adaptation is an important virulence attribute of virtually all pathogenic microbes. Understanding the regulation of iron acquisition genes will extend our knowledge of the complex regulatory changes during the infection process and might identify new potential drug targets. Thus, there is a need for efficient modelling approaches predicting key regulatory events of iron acquisition genes during the infection process.

**Results:**

This study deals with the regulation of *C. albicans *iron uptake genes during adhesion to and invasion into human oral epithelial cells. A reverse engineering strategy is presented, which is able to infer regulatory networks on the basis of gene expression data, making use of relevant selection criteria such as sparseness and robustness. An exhaustive use of available knowledge from different data sources improved the network prediction. The predicted regulatory network proposes a number of new target genes for the transcriptional regulators Rim101, Hap3, Sef1 and Tup1. Furthermore, the molecular mode of action for Tup1 is clarified. Finally, regulatory interactions between the transcription factors themselves are proposed. This study presents a model describing how *C. albicans *may regulate iron acquisition during contact with and invasion of human oral epithelial cells. There is evidence that some of the proposed regulatory interactions might also occur during oral infection.

**Conclusions:**

This study focuses on a typical problem in Systems Biology where an interesting biological phenomenon is studied using a small number of available experimental data points. To overcome this limitation, a special modelling strategy was used which identifies sparse and robust networks. The data is augmented by an exhaustive search for additional data sources, helping to make proposals on regulatory interactions and to guide the modelling approach. The proposed modelling strategy is capable of finding known regulatory interactions and predicts a number of yet unknown biologically relevant regulatory interactions.

## Background

One task in Systems Biology is to infer and model gene regulatory networks. The ultimate aim is to identify the underlying regulatory events of a system as a response to external stimuli. Thus, the regulators, their target genes and the mode of interaction need to be determined. Network inference reverse engineers regulatory networks with the help of high-throughput data and has been successfully applied in a number of studies ranging from immune diseases [1,2], full genomic models of *Escherichia coli *[3] and *Saccharomyces cerevisiae *[4,5] to models of pathogenic fungi [6]. Various approaches have been proposed for this task such as Bayesian Networks [7-9], models based on information theory [3,10,11], regression based models [1,5,12], and differential equation models [2,6,13-15]. It has been shown that the integration of different data sources improves the result of the inference approach [1,9,16,17]. Thus, several tools exploit different kinds of data within their reverse engineering algorithm [1,6,9,18]. Few regulatory models for the infection process of human pathogenic bacteria have been suggested [19-22], while there exists only one model dealing with a human pathogenic fungus [6]. Modelling gene regulatory networks of pathogenic fungi is hampered by the small number of annotated gene functions, the small number of known gene regulatory interactions and the fact that many transcription profiles focus on complicated infection processes. In contrast to defined laboratory conditions, multiple environmental parameters (e.g. pH, temperature, nutrients, CO_2_filevels) change during infection. Each parameter leads to changes in the gene expression profiles, making it difficult to conclude which environmental change leads to which effect. Furthermore, superposed and secondary effects are likely. In this paper we will focus on one specific aspect during the infection process and propose the first computational model of the regulation of iron acquisition when *C. albicans *is in contact with and invades into oral epithelial cells.

*C. albicans *is a harmless commensal yeast living in many warm blooded animals [23]. However, the fungus can change its behaviour to an aggressive pathogen within immunocompromised patients or in individuals with disrupted homeostasis of the host microbial flora [24]. Commonly, patients suffer from superficial mucusal infections, but the fungus is also able to enter the bloodstream and to cause systemic infections with high mortality rates [25]. The number of infections has dramatically increased within the last decades, mainly because of a growing number of susceptible individuals (AIDS, organ transplantation, major surgery and chemotherapy patients) [26,27]. Strikingly, *C. albicans *is able to adapt to a wide range of environmental changes such as pH, nutrient shift and temperature, and can infect virtually every human organ [25]. During infection, the fungus is able to reversibly change its growth form from an ovoid yeast growth form to elongated pseudohyphal and hyphal growth forms. This so called yeast-hypha transition has been shown to be an important virulence trait because hyphae are able to actively penetrate and destroy tissue [24]. Adherence, invasion and destruction of different human tissue are important virulence attributes of *C. albicans*. Other important virulence factors are genes involved in the interaction with cells of the immune system as well as genes involved in nutrient acquisition, stress response, and interaction with other host cells [24,28,29].

Iron is an essential mineral required as a cofactor for several proteins, as well as for a number of biochemical processes. However, within the human host, iron is bound to storage proteins such as haemoglobin, ferritin, transferrin, and lactoferrin. Consequently, there is almost no free iron available [30]. Thus, the acquisition of this mineral is an important virulence attribute of most pathogens. The importance of an effective and robust iron uptake system in *C. albicans *is indicated by three facts: First, the *C. albicans *genome contains more iron acquisition genes than that of the non-pathogenic yeast *Saccharomyces cerevisiae*. Second, colonization, as well as proliferation, are only possible if sufficient amounts of this mineral are accessible [31]. Third, mutations of certain genes involved in iron uptake increase the sensitivity of the pathogen to antifungal drugs [32,33] or reduce the virulence of the pathogen [34].

*C. albicans *exhibits at least three different iron uptake systems reflecting the possibility of acquiring iron under very different environmental conditions (reviewed in [31,35]). One possible iron source are siderophores, small iron chelating compounds with a high affinity for the mineral, secreted by microorganisms. Even though there seem to be no genes coding for siderophore biosynthesis factors [36], *C. albicans *is able to use siderophores produced by other microorganisms. One transporter of ferrichrome siderophore uptake has been identified [37,38]. Haemoglobin can also be exploited as an iron source [39]. Both, a haemoglobin-receptor gene family and a gene coding for a heme-oxygenase have been identified [40,41]. To utilize iron from transferrin, ferritin or the environment, *C. albicans *uses a high affinity reductive pathway consisting of three steps. In the first step insoluble extracellular ferric iron (Fe^2+^) is reduced to its soluble ferrous form (Fe^3+^). The *C. albicans *genome codes for 17 putative ferric reductases, which are able to facilitate this reduction [35]. In the second step, toxic ferrous iron is oxidised to ferric iron via five potential multicopper oxidases. In the last step, iron permeases form a complex with the multicopper oxidases and transport ferric iron into the cell. Four putative permease genes exist in the *C. albicans *genome [35]. Given the fact that the fungal genome codes for a set of similar proteins which are putatively able to perform each single step of iron uptake, it is of high interest to study which proteins are used during invasion of epithelial cells at which time.

*In vitro *studies have identified a number of genes and regulators involved in the response of *C. albicans *to limited iron [36]. Rim101 is a transcription factor involved in alkaline pH response [42,43]. However, transcript profiling of *RIM101 *knockout mutants also revealed a number of differentially expressed iron acquisition genes [44]. Furthermore, the ferric reductase genes *FRP1 *and *FRE2 *are directly regulated by interaction with Rim101 [45,46]. Tup1 is a general transcriptional co-repressor which is involved in the regulation of reductive iron uptake [47]. Several iron transport genes were differentially expressed upon deletion of *TUP1 *[48]. However, Tup1 may not bind directly to DNA and its mode of interaction remains unclear. Pelletier and co-workers identified Sfu1 as a suppressor of iron uptake which is able to specifically bind to DNA [49]. Furthermore, they show direct interaction of *S. pombe *Tup1 and *C. albicans *Sfu1fileading to the hypothesis that Sfu1 is the DNA binding protein which recruits the general co-repressor Tup1 to the promoters of iron acquisition genes (as shown in *Schizosaccharomyces pombe *[50]). This idea is further referred to as the "Sfu1-Tup1 hypothesis". In a phenotype study of all transcription factors of *C. albicans *, the regulator Sef1 was shown to be involved in controlling the expression of iron acquisition genes [51]. However, the molecular action of Sef1 still remains unclear. Baek *et al*. demonstrated that CBF transcription factors are involved in chelator mediated induction of *FRP1 *expression via specific DNA binding [46]. Although there exists some knowledge about the regulation of iron acquisition genes *in vitro*, almost nothing is known about their regulation when *C. albicans *is adhering to and penetrating into epithelial cells.

In this study we propose the first computational model of the regulation of iron acquisition genes in *C. albicans *using high-throughput gene expression time series data during contact with and invasion into human oral epithelial cells [52]. Our modelling approach is based on linear differential equations and utilizes selection criteria such as sparseness and robustness [53]. The integration of different data sources has been shown to improve the reverse engineering approach [1,9,54]. Hence, our model softly integrates three kinds of prior knowledge: Transcription factor binding sites [46,49,55], in vitro expression data under limited iron [36], as well as analysis of transcription factor knockout mutants [46-49]. The final model consists of a number of gene regulatory relationships. Some of them are validated by literature, while others reveal yet unknown biological relevant interactions.

## Results and Discussion

### Differentially expressed genes during contact to and invasion of oral epithelium

With the aim of modelling, candidate genes were filtered from the set of differentially expressed and by using overrepresented GO categories. Data preprocessing identified 1382 genes which were differentially expressed during experimental RHE infection at at least one point in time using an adjusted p-value cut-off of 0.05. Gene ontology (GO) processes enrichment analysis was used in order to identify key biological processes most significantly enriched with differentially expressed genes during adherence to and invasion into human epithelial cells (see additional file 1). Examples for significantly enriched categories are "pathogenesis", "fungal-type spore wall assembly", "adhesion to host". This shows that the definition of differentially expressed genes is capable of identifying biologically relevant genes, i.e. genes involved in processes relevant to virulence. The GO category "iron ion transport" was most significantly enriched with genes differentially expressed during experimental RHE infection. Fifteen out of 29 genes annotated to this process were differentially expressed. Even though most GO category annotations are based on sequence similarity to the distantly related baker's yeast *S. cerevisiae*, this high number of differentially expressed iron acquisition genes suggested that this process is important during interaction with human oral epithelium.

**Figure 1:**
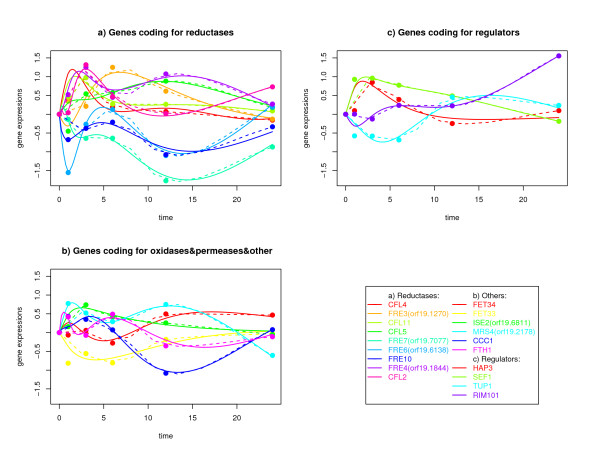
**Measured, interpolated and simulated expression time courses**. The figure shows gene expression time courses of iron acquisition genes and their regulators. Dots, measured values; dashed lines, interpolated time courses; solid lines, time courses simulated by the inferred regulatory model. (a) Nine reductase genes; (b) oxidase genes, permease genes and genes coding for inner membrane transporters; (c) genes coding for regulators.

The *C. albicans *genome codes for at least 17 putative ferric reductases able to perform the first step of iron uptake via the high affinity reductive pathway. The functional annotations for only three of these corresponding genes have been experimentally validated: *CFL1*, *FRE10*, *FRP1 *[45,56,57]. During experimental RHE infection nine genes putatively coding for ferric reductases were differentially expressed. The dynamics of expression levels of these genes varies significantly, suggesting that the fungus uses different specific reductases under different conditions (see Figure 1 Part (a)). The functional annotation of these nine proteins is based on sequence similarity to *S. cerevisiae*. This study supposes for the first time that those genes are used by *C. albicans *during interaction with epithelial cells.

Of five genes potentially coding for ferric oxidases, two were differentially expressed during experimental RHE infection: *FET33 *and *FET34*, which were also up-regulated under *in vitro *limited iron conditions [36]. The dynamics of expression levels of the two genes were similar, although *FET34 *was slightly higher expressed (Figure 1 Part (b)).

*FTH1*, a gene coding for a ferric permease, is up-regulated early during the first hour of experimental RHE infection (Figure 1 Part (b)).

There are three further differentially expressed genes annotated to the GO category "iron transport": The first, orf19.2178, codes for an ortholog of Mrs4 in *S. cerevisiae*. Interestingly, this protein is directly involved in mitochondrial iron uptake under conditions when this mineral is limited [58]. The second, orf19.6811, codes for a protein which is similar to Ise2, a member of the Fe/S cluster biosynthesis machinery of the mitochondrial matrix in baker's yeast [59]. The third gene, *CCC1*, codes for a putative Fe^2+^/Mn^2+ ^transporter which mediates vascular iron storage and is thus important to control the cytosolic iron level [60].

During the adherence to and penetration into epithelial cells, the fungus is faced with a number of environmental changes. To adapt to new environments, *C. albicans *dramatically alters its regulatory program. This is demonstrated by a total number of 67 differentially expressed genes annotated to the GO category "transcription regulator activity". A number of regulators have been identified to control the expression of iron acquisition genes (see Background). The genes *RIM101*, *HAP3*, *SEF1 *and *TUP1*, coding for transcriptional regulators, are differentially expressed during experimental RHE infection (figure 1 Part (c)) and thus used as candidate genes within the modelling approach. Another regulator which has been shown to be involved in suppressing iron transport genes is Sfu1 [49]. However, *SFU1 *it is not differentially expressed during experimental RHE infection. Possible explanations for this fact might be that the gene is transiently expressed, or the transcription factor activity might be regulated at the protein level. This study tests the "Sfu1 - Tup1 hypothesis" (see Background, Methods). This means that the molecular mode of Sfu1 is indirectly modelled (hidden in the edges starting from Tup1).

### Time course of iron limitation

**Figure 2:**
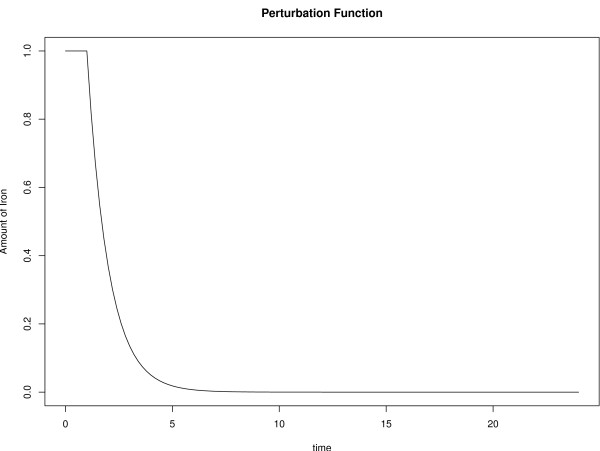
**Perturbation function**. The figure visualises the perturbation function used to model the limitation of iron during the infection process. After a delay of 60 minutes the iron avaibility is modelled by an exponential loss.

The used tool for regulatory network inference is based on differential equations and models the expression of a gene at a specific timepoint as the weighted sum of the expression of all other genes and an external stimulus (perturbation function see Methods). In this study the perturbation function models the decreasing amount of iron the fungus is faced with during experimental infection. As it is unknown how the availability of iron for *C. albicans *changes over time during the experimental RHE infection, different types of perturbation profiles describing the iron availability were tested. A decreasing amount of the mineral was used as the environmental stimulus of the network model, reflected by the perturbation function *u*(*t*) (see Methods). Network models were predicted for fourteen different types of perturbation functions and the model-error, as well as the data-error were compared (see additional file 2). Best results were achieved with an exponential decrease of iron during experimental RHE infection. Furthermore, there was a slight decrease of the data-error if the early availability of iron was modelled with a decrease after a certain delay. Optimal values were achieved by using a constant iron concentration until 60 minutes followed by an exponential decrease of iron (see Figure 2). The model-error is also minimal for this perturbation function, which therefore was used in the final model. The data-error and the model-error increase again when using a 90 minutes delay. It seems that *C. albicans *is not exposed to higher levels of iron within 24 hours post infection, since a perturbation function with a growing amount of iron generates a higher data and model error.

### Regulatory network of iron acquisition genes

**Figure 3:**
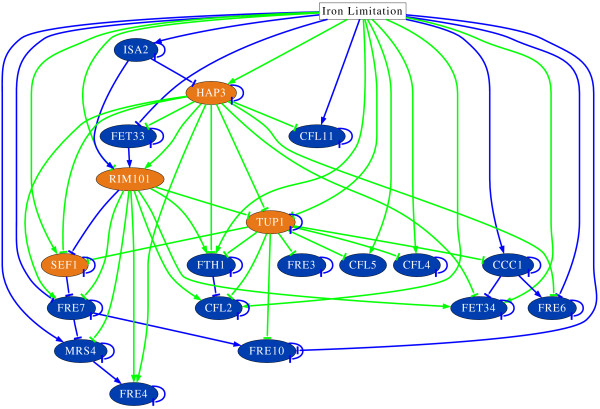
**Inferred regulatory network model**. The inferred regulatory network model. Arrow, activating interaction; bar, repressing interaction. Green edge, consistent with prior knowledge; blue edge, newly predicted edge. Edges resampled less than 50% times are neglected.

A regulatory network model was inferred which is based on differential equations and an exhaustive list of prior knowledge based on other data than time series gene expression data (see Methods). Figure 3 presents this regulatory network model (see also additional file 3). The model consists of fifteen differentially expressed iron acquisition genes and four differentially expressed regulators. The modelling approach found a sparse network with 63 edges. The differential equation model is still able to give a good fit to the measured time series data (see Figure 1). The initial model fits to the time series data with a data-error of 0.105 and to the prior knowledge with a model-error of 0.330. To indicate which edges of this model are robust to random fluctuations, the time series data were perturbed and the modelling approach was iterated 1000 times (see Methods). Perturbing the time series data causes only a small change of the quality of the inferred networks, which is quantified by a mean data-error of 0.237 with a variance of 0.007. Regulatory interactions which are robust against perturbing the time series data are also robust against changing parts of the prior knowledge. Thus, the cross-validation of the prior knowledge (see Methods) resulted in four interactions which were found more than 50% in the random perturbed models but less than 50% in the cross-validation models. Six interactions not predicted by the initial model were predicted more than 50% in all models predicted by the cross-validation of prior knowledge and perturbation of time series. When increasing the cut-off, which defines an edge to be stable, the number of inferred edges decreases in a nearly linear way (data not shown). The most stable edges are those with the highest score in the prior knowledge. Furthermore, regulatory influences from the perturbation proove that they are very stable.

The proposed modelling approach is capable of identifying biological meaningful interactions, which is illustrated by the rediscovery of already known transcription factor target genes. For example, the inferred regulation of *CFL2 *by Rim101 and *FRE10 *by Tup1 has been shown with the help of *in vitro *EMSA and Northern Blots [46,47]. Furthermore, the model provides the first evidence that these interactions take place during experimental RHE infection.

A number of further interactions predicted by the prior knowledge are found in the final model. There are twelve interactions in the model which were predicted by *in vitro *expression studies (source 2). This study adds first evidence of these regulatory interactions taking place when *C. albicans *adheres to and invades into epithelial cells. A total of 22 interactions predicted by the occurrence of TFBS (source 3) can be found in the model. Seven of these 22 putative interactions are additionally predicted by source 2 or source 1 while 15 are exclusively predicted by source 3. Since there is no former evidence in the literature, this is the first time that these genes are predicted to be target genes of the respective transcription factors (see also table 1).

**Table 1:** Transcriptional regulators and their predicted target genes

Regulator source	Prior 1/2/3	found from prior 1/2/3	newly predicted target genes
Hap3	0/0/15	0/0/10	*CFL11*, *FTH1*, *SEF1*, *TUP1*, *HAP3*, *FRE7 *(orf19.7077), *FET33 FRE4 *(orf19.1844), *FET34*, *FRE6 *(orf19.6138), *RIM101*

Rim101	2/4/7	2/1/6	*FRE4 *(orf19.1844), *MRS4 *(orf19.2178) *TUP1*, *FTH1*

Tup1	1/7/14	1/6/5	*FRE3 *(orf19.1270),*TUP1*,

Sef1	0/0/0	0/0/0	*FRE7 *(orf19.7077)

For each regulator the number of compiled prior knowledge edges, the number of inferred edges consistent with this prior knowledge, and newly predicted target genes are shown. For description of the three prior knowledge sources see Methods. A target gene is scored as "newly predicted" if it was predicted by the time series data without prior knowledge, or if it was predicted by prior knowledge based on the occurrence of TFBS (source 3) and was found to be consistent with the time series data. Newly predicted target genes are ordered in respect to the stability of the regulatory interaction (number of re-sampling during random perturbation of time series data and cross-validation of prior knowledge).

The inferred network model shows a hub like structure. Given a number of potential transcription factor - target gene interactions proposed by the prior knowledge, those interactions are preferred in the final model. Hap3 regulates eleven target genes, Tup1 eight and Rim101 regulates seven genes. This study gives the first proposal of a target gene for the transcriptional regulator Sef1: *FRE7 *(orf19.7077). In most cases there are two evidences for these regulations to happen during the infection process. For example, the regulation of *FTH1 *by Rim101 is supported by the gene expression time series during RHE infection and the occurrence of the Rim101 binding site in the upstream region of *FTH1*. In these cases the presented model predicts direct physical transcription factor - target gene interaction. Even though they have a small score in the prior knowledge (source 3), regulatory interactions of Hap3 prove very stable against random perturbation of the time series data and cross-validation of prior knowledge. In a network inferred without any prior knowledge, five interactions of Hap3 are consistent with those proposed in the final model. All proposed target genes of Tup1, but not *SEF1*, have the Sfu1 binding site in their upstream region. This adds further evidence to the "Sfu1-Tup1 hypothesis".

The model also provides suggestions of how the transcriptional regulators may be regulated. In most cases an influence by the external stimulus (i.e. limited iron) is predicted, which might indicate regulations at protein level. However, Hap3 might be involved in the transcriptional activation of *RIM101*, predicted by the occurrence of the Hap3 binding site in the *Rim101 *promoter and the time courses of both genes. Furthermore, Hap3 and Rim101 seem to inhibit the expression of the gene coding for the general repressor Tup1. In this way Hap3 and Rim101 have an additional indirect positive influence on the transcriptional rate of iron acquisition genes by inhibiting their repressor. It is known that the repression of hub-like nodes increase the stability of a system [61]. Table 1 summarises all predicted and inferred target genes of the transcriptional regulators in this study.

Even though transcriptional regulator - target gene interactions are preferred by using the prior knowledge, the model also predicts indirect regulatory influences of one gene to another. For example, the gene coding for the putative mitochondrial transporter Mrs4 (orf19.2178) has a negative influence on the gene coding for the reductase *FRE4 *(orf19.1844). This might happen in cases where sufficient amount of iron is available and the fungus pumps it into the mitochondria.

There are 16 edges predicted by the prior knowledge which contradict the time series expression data or are not found to be robust and are thus not present in the proposed model. For example the regulation of *FRE7 *(orf19.1270) by Rim101 was predicted under *in vitro *alkaline conditions but does not seem to occur during experimental RHE infection. Furthermore, the modelling approach identified a number of 31 regulatory events which are not present in the prior knowledge. These are 16 self degradations, eight influences from the external stimulus, and seven gene- gene influences.

The proposed regulatory network model consists of 19 differentially expressed genes during experimental oral infection, which were chosen because they are involved in the important process of iron acquisition. However, there is a number of 1363 remaining differentially expressed genes which are not covered by the model. With the help of fuzzy c-means clustering all differentially expressed genes were grouped into six significant time profiles (see Methods). Each cluster profile is characterised by having an extremum at one of the measured time points (additional file 4). For each profile, one of the genes of the proposed regulatory model can be considered as a profile-representative. In this way regulatory influences inferred by our model can be transferred to other pairs of genes within the respective profiles. The profiles contain a number of genes with so far unknown function. With the knowledge of co-expression patterns and the proposed regulatory influences in our model it is possible to infer putative functions for these genes.

This study used expression time series data from an experimental RHE infection. This experimental infection covers some important aspects of the oral infection process, such as the adherence to epithelial tissue, the yeast-hypha transition, tissue penetration, pH shift and limited iron. On the other hand, further important aspects are missing, such as the interaction with immune system cells and other microorganisms. The proposed modeling approach focuses on the regulation of iron acquisition genes. Some of the proposed regulatory interactions might also happen during oral infection. The model proposes for the first time that Rim101 directly activates *FTH1 *and *FRE4 *(orf19.1844) and indirectly activates *CFL2 *and *CFL5 *via repressing the gene coding for their repressor Tup1. These predictions are supported by the occurrence of the respective TFBS in the upstream regulatory regions and the time series data. Those four genes and *RIM101 *are also up-regulated at least two-fold in an expression data set of patients suffering from oral candidiasis [52]. This implies that those regulatory interactions might also happen during real oral infections.

During experimental RHE infection, *C. albicans *is faced with a rapidly changing environment. The final gene expression pattern results from all these changing environmental parameters. A perturbation function was used, which models the limitation of iron. However, it remains unclear if the final gene regulatory interactions are a result of iron limitation or other changing environmental parameters. For instance, the transcription factor Rim101 is also involved in alkaline pH response, i.e. an environmental factor which also changes during the infection process. In a follow-up experiment the expression profile of *C. albicans *in rich medium could be compared to the expression profile in a medium without iron. By performing *in vitro *iron limitation time series expression experiments the data could be used to clarify which of the proposed regulatory interactions in this study are only due to limited iron. These interactions could then be validated in further experiments. The proposed model here consists of a number of influences from the external perturbation whose molecular action remains unclear. With the help of *in vitro *iron limitation expression data it may be possible to identify so far unknown regulators whose role in iron acquisition as well as for virulence may be studied in the future. Together with the proposed regulatory model in this study, which focuses on an infection condition, novel virulence factors may be identified.

A well known problem when using differential equation models with a high number of parameters is over-fitting. Equation 1 consists of a large number of parameters while there are only five measured points in time. A model which uses all parameters would clearly over-fit the measured data. To overcome this problem the proposed modelling approach minimises the number of non-zero parameters by using a search strategy. Furthermore, the soft integration of prior knowledge guides the inference approach to a knowledge-driven solution. Finally, by disturbing the time series data and repeating the inference approach, parameters which are robustly unequal to zero were identified. Another way of coping with over-fitting is cross-validation (e.g. leave one out). This strategy was not used in this study because skipping parts of the rare measured data would disrupt the ratio between parameters and data points even further. Another general limitation is that differential equation models assume the system to be in a steady state before the experiment. Since *C. albicans *was grown on rich media before the actual experiment was performed this assumption is valid here.

## Conclusions

This study focuses on a typical problem in Systems Biology where an interesting biological phenomenon is studied by a small number of experimentally available data points. To overcome this limitation a special modelling strategy is applied: First of all, the modelling approach searches for the most important features, variables and structure able to model the measured kinetics. Second, random perturbation of the input data is used to infer robust regulatory interactions. Finally, different data sources other than time series expression data were used to overcome the data limitation. The present study uses three heterogeneous data sources to compile an exhaustive list of prior knowledge which is softly integrated into the modelling approach and thus guides the network prediction to a knowledge assisted solution. With the help of this strategy a number of biologically relevant gene regulatory interactions were predicted, even in the case of a limited amount of data. The strategy of using prior knowledge to overcome identification problems arising from a small amount of data could be used in many Systems Biology application.

This study focused on one particular process of *C. albicans *during contact with and invasion into human oral epithelial cells: the regulation of iron acquisition genes. Initially it proposed fifteen iron acquisition and four genes coding for regulators which *C. albicans *activates during experimental RHE infection. Furthermore, a network model is proposed consisting of 63 regulatory relationships during experimental RHE infection process. Some of them have already been found in in other *in vitro *studies. This confirmation demonstrates that the employed inference approach is capable of identifying biologically relevant interactions. A number of further yet unknown interactions were predicted. Especially, a number of further target genes for transcription factors involved in regulating iron acquisition genes were predicted. The model predicted four new target genes of Rim101. Additionally, three potential target genes predicted by *in vitro *analysis of knockout mutants were also regulated by Rim101 during experimental RHE infection. The first target gene of Sef1 was predcited: *FRE7 *(orf19.7077). Eleven target genes were newly predicted to be regulated by Hap3. Further evidence was found for the supporting the hypothesis that Sfu1 is the DNA binding protein recruiting Tup1 to the promoters of iron acquisition genes ("Sfu1 -Tup1 hypothesis"). One gene was newly predicted to be regulated this way (*FRE3 *(orf19.1270)). Five potential target genes of Tup1, which were already predicted by expression analysis of knockout mutants, were now predicted to be also regulated during experimental RHE infection by the Sfu1 - Tup1 complex. Very interestingly, potential regulations of the transcription factors are proposed. Hap3 is involved in the regulation of the gene coding for Rim101 and Tup1. Rim101 and Hap3 repress *TUP1 *during RHE infection. In this way the two regulators activate iron acquisition genes also indirectly by repressing their transcriptional repressor. There is evidence that some of the proposed regulatory interactions might also happen during oral infection. The gene coding for Rim101 and three (in)direct target genes (*FTH1*, *FRE4 *(orf19.1844), *CFL2*,) proposed by the model were also up-regulated within expression data of patients suffering from oral candidiasis.

In follow-up experiments it will first be necessary to determine which of the proposed regulatory interactions are due to other changing parameters during infection process than iron limitation. The remaining interactions could then be experimentally validated. This would lead to more detailed insights into the mechanisms of how pathogens regulate important processes, such as iron acquisition, during infection processes. These insights could be transferred to other pathogenic organisms, especially closely related pathogenic fungi such as *C. dubliniensis *or *C. tropicalis*. Moreover, with the help of clustering results, it is also possible build hypotheses of regulatory influences between genes with so far unknown function.

## Methods

### Data

Zakikhany *et al*. [52] performed genome wide transcript profiling of *C. albicans *during experimental infection of reconstituted human oral epithelium (RHE). Gene expression was monitored at five points in time (1 h, 3 h, 6 h, 12 h, 24 h post infection) with two to four biological replicates. Each array contained two replicated spots for each gene. The relative mRNA expression of each point in time was compared to the mRNA expression of *C. albicans *grown on YPD (rich) medium (referred to as common reference). Additionally, Zakikhany *et al*. [52] performed expression studies of *C. albicans *cells from eleven patients with oral candidiasis. GenePix files and raw imagefiles were downloaded from http://www.galarfungail.org/data.htm.

### Preprocessing and clustering

Data was preprocessed using the Limma package [62] of the statistical language "R" [63]. Two arrays were removed from further analysis because of low correlation of replicated spots on these arrays (third replicate after 3 h, fourth replicate after 24 h). "Lowess" ' normalisation was used to correct for spatial effects or cross-hybridisation on each array. The logarithmic fold-change (logFC) comparing expression values during infection with the common reference was calculated. "Quantile" normalisation was used to ensure that log ratios have the same empirical distribution across arrays which facilitates between array comparisons. Empirical Bayes statistics [64] were applied to scan for significantly differentially expressed genes at every point in time making use of both replicated microarray experiments and intra - array replicated spots [65]. The false discovery rate was controlled with help of the "Benjamini and Yekutieli" [66] correction method. An adjusted p-value cutoff of 0.05 was used to define differentially expressed genes. In case of patients data genes were defined to be up-regulated if they were at least two-fold higher expressed than the common reference. Gene expression profiles of all differentially expressed genes during experimental oral infection were clustered using fuzzy c-means clustering [67]. The number of clusters was estimated as previously described [2].

### Over-represented Gene Ontology categories

Gene Ontology(GO) [68] category over-representation was used to identify key biological processes most significantly enriched with differentially expressed genes during experimental RHE infection. GO annotation data were downloaded from the "*Candida *Genome Database" [69]. A well known problem in studying GO category over-representation is the fact that some general parent categories are only over-represented because their more specific children categories are significantly enriched with differentially expressed genes. To overcome this limitation we focused on the most specific over-represented GO categories using the "weight" algorithm of the package "topGO" [70]. In short, this method uses weights based on the score of neighbouring nodes in the GO graph. To decide whether a parent category better represents the set of differentially expressed genes than its children categories the enrichment score of the parent category is compared to that of the children categories in a bottom up manner. If the children have a higher score their corresponding genes receive a smaller weight in the parent category. The weights are finally used in the statistic test. In this study Fisher's exact test was applied and a p-value cutoff of 0.05 was used. Genes annotated to the most significantly enriched GO process "iron ion transport" were used as candidate genes for the modelling approach. The list of candidate genes was augmented by genes encoding for differentially expressed regulators which are known to be involved in regulating iron acquisition genes: Hap3, Rim101, Tup1 and Sef1 (see background).

### Network prediction

The Net*Gene*rator tool [53] was used to predict gene regulatory networks. This tool has been successfully applied to model gene regulatory networks based on transcript profiling time series data of globally perturbed organisms [6]. Net*Gene*rator is based on differential equations and models the expression of gene *i*(*i *= 1..*n*) at time *t *as the weighted sum of the expression of all other genes and an external stimulus (iron limitation) at time *t*. Based on the given time series data, the tool calculates the gene regulatory matrix *W *and the perturbation vector *B*. The parameter *w_i, j _*(component of *W*) represents a regulatory interaction between the two genes *i *and *j *while the parameter *b_i _*(component of *B*) represent an influence from the external stimulus given by the function *u*(*t*) on gene *i *(see equation 1). Non-zero parameters define the regulatory network. Therefore, a positive parameter denotes an activation and a negative parameter denotes a repression. Regulatory interactions inferred by this model do not necessarily reflect direct physical interactions. In fact, the model may also infer indirect regulatory influences mediated by one or several molecular reactions.

(1) $${\dot{x}}_{i}(t)=\sum_{j=1}^{n}w_{i,j}x_{j}(t)+b_{i}u(t)$$

One key property of gene regulatory networks is structural sparseness which relates to the fact that there are less edges in gene regulatory networks than expected from a random network [71]. The Net*Gene*rator tool is specifically designed to infer sparse regulatory networks. This is achieved by separating the optimisation of the model structure from the optimisation of the parameters. Thus, the tool tries to maximise the number of parameters equal to zero while still being able to fit to the observed gene expression time courses. The model structure is identified with the help of a heuristic search strategy. For each potential model structure non-zero parameters are optimised by standard mathematical algorithms subjected to minimise the error function for each time series given by the quadratic error to the measured data (see equation 2).

(2) $$Err=\sum_{i=1}^{n}\sum_{k=1}^{T}{\left({\hat{x}}_{i,k}-x_{i}(t_{k},W,B)\right)}^{2}$$

Searching for an optimal model structure is supported by the integration of knowledge based on different sources than gene expression time series. The structure optimisation procedure is assisted by giving proposals for the gene regulatory matrix *W*^*Prior *^and for the perturbation vector *B*^*Prior*^. There are four types of proposed regulatory interactions: (a)ctivation, (r)epression, (i)nteraction, (n)o interaction. For an (i)nteraction it is not known whether it is a repressing or an activating regulation. This so called "prior knowledge" $\left(w_{i,j}^{Prior},b_{i}^{Prior}\epsilon \{a,r,i,n\}\right)$ is softly integrated into the modelling approach, i.e. a regulatory interaction given by the prior knowledge only remains in the final model if it fits to the observed expression data. The confidence of each putative interaction given by the prior knowledge is given by the scores *β^W ^*for the gene to gene influence and scores *β^B ^*for the stimulus- gene influence. The analysis of the time series data and the prior knowledge is performed at the same time in parallel. Mathematically this is modelled by an additional summand in the error term for each time series (see equation 3). The first part of this error term is the same as in equation 2 and is used to optimise the model in respect to the measured time series data. The second part optimises the model in respect to the given prior knowledge. In case of differences between the inferred model and the prior knowledge but a good fit to the time series data the high model-error can be balanced out by a small data-error. The global parameter *λ *weights the influence of the model-error in equation 2.

(3) $$\begin{array}{l} \;\;Err=\underset{Data-error}{\underset{︸}{\sum_{i=1}^{n}\sum_{k=1}^{T}{\left({\hat{x}}_{i,k}-x_{i}(t_{k},W,B)\right)}^{2}+}}\\ \;\;\underset{Model-error}{\underset{︸}{\lambda \underset{Unweighted-model-error}{\underset{︸}{\left(\sum_{i=1}^{n}\sum_{j=1}^{n}\beta_{i,j}^{W}*d_{i,j}^{W}+\sum_{j=1}^{n}\beta_{i}^{B}*d_{i}^{B}\right)}}}}\\ d_{i,k}^{W}=\{\begin{array}{ll} 0 & {\hat{w}}_{i,k}==w_{i,k}^{Prior}\\ 0 & {\hat{w}}_{i,k}==(r\vee a)\wedge w_{i,k}^{Prior}==i\\ 1 & otherwise \end{array}\\ \;d_{i}^{B}=\{\begin{array}{ll} 0 & {\hat{b}}_{i}==b_{i}^{Prior}\\ 0 & {\hat{b}}_{i}==(r\vee a)\wedge b_{i}^{Prior}==i\\ 1 & otherwise \end{array}\\ \;\;\;\;{\hat{w}}_{i,k}=\{\begin{array}{cc} a & sign(w_{i,k})=1\\ r & sign(w_{i,k})=-1\\ n & sign(w_{i,k})=0 \end{array}\\ \;\;\;\;\;\;{\hat{b}}_{i}=\{\begin{array}{cc} a & sign(b_{i})=1\\ r & sign(b_{i})=-1\\ n & sign(b_{i})=0 \end{array} \end{array}$$

#### Prior knowledge

Several studies demonstrated that integrating several data sources improves the reverse engineering approach [1,9,54]. Since different data sources might be contradictory, it is advantageous to softly integrate them during the modelling procedure [54,72]. It is important to note that interactions proposed by the prior knowledge alone might not be sufficient to adapt to the measured time series data. In this case the inference approach is also allowed to add further regulatory influences not proposed by the prior knowledge. The proposed inference approach softly integrates 51 putative gene regulatory influences extracted from different data sources (see additional file 5). Three different sources are used to compile prior knowledge for the prediction of gene regulatory networks:

**Source 1: **Analysis of transcriptional regulator knockout mutants and direct experimental verification of physical transcriptional regulator - target gene interactions (EMSA, RT-PCR, Northern blot).

**Source 2: **Gene expression studies under limited iron conditions and expression analysis of transcriptional regulator knockout mutants.

**Source 3: **Occurrence of transcription factor binding sites (TFBS) in the upstream intergenic regions of iron acquisition genes.

The following information was used to compile prior knowledge from source 1: Four differentially expressed transcription factors have been shown to be directly involved in the regulation of iron acquisition genes via phenotype analysis of knockout mutants: Rim101 [45,46], Hap3 [46], Tup1 [47,49], and Sef1 [51]. For these factors, an influence from the external stimulus (limited iron) is assumed. With the help of electronic mobility shift assays (EMSA), Beak *et al*. found that *CFL2 *is regulated by Rim101 but not by Hap3 [46]. Furthermore, real time PCR was used to identify the repression of orf19.7077 (*FRE7*) by Rim101 [73]. Finally, the regulation of *FRE10 *(*CFL95*) by Tup1 was demonstrated with the help of Northern blots [47]. Taken together, three regulator - gene interactions and four stimulus - gene influences were extracted from source 1.

Eleven regulator - gene interactions and five influences from the external stimulus were predicted with the help of source 2. Lan *et al*. compared relative gene expression of *C. albicans *under conditions where iron is limited with conditions where sufficient amount of the mineral is available [36]. In this study, five genes were significantly up-regulated under low iron conditions which are also differentially expressed in the present study: *CFl2*, *FTH1*, *FET34*, *CFL5 *and *CFL4*. For these genes, a regulator influence by the external stimulus (limited iron) is assumed. Microarray analysis compared gene expressions of transcription factor knockout mutants *rim101*Δ [44] and *tup1*Δ [48] to the respective wild type under various *in vitro *conditions. A number of genes are significantly differentially expressed in these knockout mutants. For those genes a regulation by the respective transcription factor is assumed as prior knowledge. Sfu1 is a suppressor of iron uptake which might recruit Tup1 to its target genes [49]. Since *SFU1 *is not differentially expressed during contact with oral epithelial cells (see results), the "Sfu1-Tup1 hypothesis" was tested by proposing genes which might be regulated by Sfu1 to be target genes of Tup1. There are five genes significantly up-regulated in both, a *tup1*Δ and a *sfu1*Δ mutant [36,48] and one which is up-regulated only in a *SFU1 *mutant. All six genes were proposed target genes for Tup1 in source 2.

Transcription factors regulate their target genes by specific DNA binding. Thus, knowledge of TFBS helps to identify potential target genes regulated by direct physical interaction with a transcription factor. Thus, the occurrence of TFBS in the regulatory regions of iron acquisition genes was used in data source 3.

TFBS are know for the transcriptional regulators Rim101, Hap3 and Sfu1. Rim101 binds to the 5'-CCAAGAA-3' of the ferric reductase gene *FRP1 *[45]. Hap3 is a member of the CBF transcription factors which specifically bind to 5'-CCAAT-3' sites of their target genes [46,74]. Sfu1 binds to the 5'-[A/T]GATAA-3' of iron acquisition genes and is believed to be the DNA binding protein which recruits the general co-repressor Tup1 [49]. Genomic and flanking sequences of the differentially expressed iron acquisition genes during experimental RHE infection were downloaded [69]. The 1000 basepairs upstream (from the start codon) region, which is not part of the open reading frame of another gene (intergenic region), was taken into account. Each gene which has at least one occurrence for the respective binding site (or its complementary sequence) in its intergenic upstream region is assumed to be regulated by the respective transcription factor in the prior knowledge. Genes which have the Sfu1 binding site in their upstream region are assumed to be regulated by Tup1 in order to test the "Sfu1-Tup1 hypothesis". Altogether, 36 putative regulatory interactions are predicted from the data source 3.

Each different data source used as prior knowledge for the inference procedure does not ultimately prove the existence of this interaction. In fact, there is different confidence for the different data sources. The reverse engineering approach used in this study offers the opportunity to use a score reflecting this confidence differences. Data source 1 has the highest confidence and thus receives a score of *s*_1_(*i*, *j*) = 0.5 comprising the putative regulation of gene/external stimulus *i *to gene *j*. It is important to note that mutants were tested under different environmental conditions than *ex vivo *RHE infection.

For data source 2, a score of *s*_2_(*i*, *j*) = 0.25 is used. In addition to different environmental conditions, these putative regulatory interactions could be based on indirect effects, i.e. an up-regulation of a putative transcription factor target gene may not be a direct effect of the knockout mutant but could be triggered by a signal cascade or pathway.

Given the short length of the known binding sites of the transcriptional regulators it might happen to find them in the upstream region by chance. For this reason, prior knowledge based on the occurrence of specific transcription factor binding sites in the upstream regions of iron acquisition genes (source 3) receives the smallest score of *s*_3_(*i*, *j*) = 0.125.

The different data sources can predict the same putative regulatory interaction. In this case there are several evidences of this prior knowledge. To consider this fact, the scoring system is additive with a maximum of 1 (see equation 4). The global parameter *λ *weights the influence of the model-error in the error term of the modelling approach (see equation 2). The influence of this global parameter on the quality of the model was studied by sampling *λ *values within [0..1]. Finally, *λ *= 0.125 was chosen, where the unweighted model-error is minimal and the data-error has an inflection point (see additional file 6 and additional file 7).

(4) $$\begin{array}{r} s(i,j)=min(1,s_{1}(i,j)+s_{2}(i,j)+s_{3}(i,j))\\ \beta_{i,j}^{W}=s(i,j),\beta_{i,j}^{B}=s(i,0) \end{array}$$

Where *s*(*i*, 0) represents score reflecting the influence from the external stimulus.

#### Time course of iron limitation

The perturbation function *u*(*t*) (see equation 1) describes the time course of the external stimulus. In this study *u*(*t*) models the available amount of iron during experimental RHE infection. The actual time course of iron avaibility is not known. Although the modelling approach is not able to predict the exact change of iron availability *C. albicans *is faced with during infection process, it is able to predict the mode of action for iron limitation. For example the modelling approach can predict whether the iron availability decreases linearly or in an exponential way. Fourteen different perturbation functions *u*(*t*) describing different modes of action for the iron limitation were used (see Additional file 2). These functions model the kinetics of iron availability in different ways; using for example a linear decrease, a quadratic decrease, an exponential decrease. Furthermore, functions with a decrease starting after a certain delay were tested. These functions model the fact that there might still be a certain amount of iron at the beginning of the experimental infection. Finally, a perturbation function with an increasing iron concentration at later points in time was tested. This function simulates the scenario in which *C. albicans *gains iron from the host after some infection time. The model-error and the data-error were compared for each perturbation function (see supplementary file 2).

#### Robustness of predicted regulatory interactions

Another characterisation of gene regulatory networks is structural robustness [75]. Generally, small changes in mRNA concentrations do not alter the inferred regulatory interactions. For this reason the network inference approach was augmented by randomly disturbing the input time series expression profiles. For each time series and each point in time, the relative mRNA concentrations were changed by adding noise sampled from a Gaussian distribution with mean 0 and variance 0.05. This was iterated 1000 times and the number of every inferred edge was counted. Edges resampled more than 500 times (50%) are interpreted as being stable comprising a robust network.

The proposed inference approach uses an exhaustive list of prior knowledge. Each predicted gene regulatory interaction might be simply a result of this prior knowledge. To test whether this is the case a cross-validation of the prior knowledge was performed. In detail, 10% of the prior knowledge was randomly skipped and the Net*Gene*rator tool was applied to the time series data. This was iterated 1000 times and regulatory interactions were counted as being robust against changes in the prior knowledge if they were found more than 500 times (50%).

## Authors' contributions

RG and BH directed the study. JL carried out the analyses on the data. JL wrote the manuscript supported by the coauthors. DW and BH assisted in interpretation of the results. All authors read and approved the final manuscript.

## Supplementary Material

Additional file 1 **Over-represented Gene Ontology categories**. List of over-represented Gene Ontology categories as result from the "weight" algorithm of the package "top-GO" [70]. Only the p-values of the "weight" algorithm were taken into account. Click here for file

Additional file 2 **Data-error and Model-error for different perturbation functions**. This table shows information about the different perturbation functions used in this study: The type of decrease of iron for each function, as well as a delay of iron depletion and a gain of iron is described. Data-error and model-error are shown for all perturbation functions used in this study. Click here for file

Additional file 3 **Regulatory network**. This file includes information about the inferred regulatory network. Numberrand = Number of networks which have this edge for perturbed time series data. Numbercrosval = Number of networks which have this edge for cross-validation of prior knowledge. Click here for file

Additional file 4 **Clustering result**. Result of fuzzy c- means clustering. All differentially expressed genes were grouped into six significant time profiles characterised by having an extremum at one of the measured time points (column 3). For each profile, one of the genes of the proposed regulatory model (first 19 rows) can be considered as a profile-representative. In this way regulatory influences inferred by our model can be transferred to other pairs of genes within the respective profiles. Click here for file

Additional file 5 **Prior knowledge**. This file includes all information about prior knowledge used in this study. Click here for file

Additional file 6 **Unweighted Model-error for different values of *λ***. The influence of this global parameter on the quality of the model was studied by sampling values within the range of [0,1] in steps of 0.025. This graph shows the unweighted model-error. The chosen value 0.125 is indicated in red. Click here for file

Additional file 7 **Data-error for different values of *λ***. The influence of this global parameter on the quality of the model was studied by sampling values within the range of [0,1] in steps of 0.025. The chosen value 0.125 is indicated in red. Click here for file
